# Seasonal Variation of Forest Butterfly Diversity in Tropical Lowland Nepal

**DOI:** 10.1002/ece3.71550

**Published:** 2025-06-17

**Authors:** Mahamad Sayab Miya, Subarna Shrestha, Annapurna Dhakal, Soniya Shrestha, Renuka Karki, Kriti Thapa, Nisha Simkhada, Prakriti Chataut, Pabitra Maya Tamang, Apeksha Chhetri

**Affiliations:** ^1^ Department of Biological Sciences Western Kentucky University Bowling Green Kentucky USA; ^2^ Faculty of Forestry Agriculture and Forestry University Hetauda Nepal; ^3^ Institute of Forestry Hetauda Campus, Tribhuvan University Hetauda Nepal; ^4^ Sault College Brampton Campus Brampton Canada; ^5^ Institute of Forestry Pokhara Campus, Tribhuvan University Pokhara Nepal

**Keywords:** Hetauda, Hill diversity, Lepidoptera, Nymphalidae, Pollard Walk, seasonality

## Abstract

Butterfly populations and diversity vary with the seasons due to bioclimatic factors, particularly precipitation and temperature. Their seasonality in the tropical region of Nepal has not been well studied, and climatic factors have yet to be incorporated into research. Hence, this study examined the seasonal variation of forest butterfly diversity, community composition, and the effect of precipitation and temperature on diversity in the tropical lowland of Nepal. Butterflies were sampled for a year using Pollard Walk and checklist methods. Different forms of diversity indices, similarity/dissimilarity, and indicator species analysis were performed using data from the Pollard Walk. Generalized Linear Mixed Models were employed to assess the effect of precipitation and temperature on species richness and abundance. Data from the checklist method was utilized to account for the overall species richness. A total of 115 butterfly species from six families were documented. The diversity and community composition varied significantly between the seasons, with two seasonal peaks of richness: pre‐monsoon and post‐monsoon. Species richness and abundance also varied significantly among the families. Species such as *Euthalia aconthea*, *Hypolimnas misippus* , *Jamides celeno,* and *Vanessa indica* were found to be strong indicators for particular seasons. Nymphalidae was the richest, most abundant, and most diverse family. Different families exhibited noticeable variations in diversity throughout the seasons. Species richness and abundance were positively affected by increased temperature but negatively affected by increased precipitation. The present study highlights the significance of seasonal shifts for butterfly diversity in a tropical region. The seasonality of butterflies in the study area may have also been influenced by anthropogenic activities and human‐created habitat heterogeneity, resulting in the dominance of generalist species during specific seasons.

## Graphical abstract

The butterfly diversity significantly varied between the seasons, with two seasonal peaks of richness: pre‐monsoon and post‐monsoon. Nymphalidae was the richest, most abundant, and most diverse family. The overall species richness and abundance were positively affected by increased temperature but negatively affected by increased precipitation.

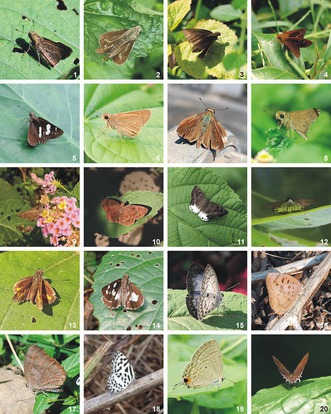

## 1 Introduction

Seasonality plays a vital role in shaping insect diversity and their populations (Silva et al. 2011). Insects are crucial components of biodiversity, covering more than half of the world's terrestrial species (Stork 2018). In tropical regions, their populations often peak during the rainy season and decline during dry periods (Wolda 1988). Butterflies are among the most charismatic and delicate insects in the order Lepidoptera, with around 19,500 described species worldwide (van Nieukerken et al. 2011; Kawahara et al. 2023). They play an inevitable role in pollination (Tiple et al. 2006) and the food chain—food for birds, amphibians, reptiles, spiders, and other insects (Tiple and Bhagwat 2023). Furthermore, they are excellent bioindicators of terrestrial ecosystems (Syaripuddin et al. 2015), as they are sensitive to habitat loss, fragmentation, disturbance, and climate change (Wilson and Maclean 2011; Ellis et al. 2019; Forsberg et al. 2020; Hill et al. 2021).

Butterflies are an ideal group to study the effects of seasonality, as some species are present year‐round while many are found only in specific seasons (Kunte 1997). Their seasonal dynamics are influenced by various factors, including air temperature, relative humidity, rainfall, natural predators, food availability, and anthropogenic disturbance factors (Silva et al. 2011; Freire et al. 2014; Habel et al. 2018). In seasonal forests, several Lepidoptera species enter a state of diapause as final instar larvae within pupal cocoons, later pupating during the following rainy season (Aiello 1992). Others may remain as pupae throughout the dry season, emerging as adults once the rain returns (Janzen 1987). Some butterflies enter reproductive diapause as adults during the dry season (DeVries 1987), while others breed continuously and adjust their geographic distributions based on the availability of larval food plants (Jones and Rienks 1987). The gradual increase in temperature is affecting insects in terms of behavior, physiology, distribution, and species interaction, as well as the increased frequency of extremes such as hot/cold, fires, drought, and floods on those parameters (Harvey et al. 2023). Hence, the recent issue of climate change and global warming could severely impact the threatened tropical butterflies (Bonebrake et al. 2016; Chowdhury 2023). Thus, studying the seasonality of butterflies and the effects of environmental factors on their diversity is essential for butterfly conservation efforts.

Nepal, a South Asian country, is divided into six climatic zones, ranging from tropical in the southern plain to tundra/nival in the north with snow cover (Paudel et al. 2021). The diverse climate and geography of the country host about 695 described species of butterflies (Van der Poel and Smetacek 2022; KC et al. 2025). However, they are sparsely studied across different climatic regions and seasons in Nepal, with many existing studies focused on subtropical to temperate areas. For example, from these zones, butterfly diversity in various habitats and seasons has been studied by scholars such as Thapa (2008), Khanal et al. (2012), Khanal (2020), Miya et al. (2021), and Neupane and Miya et al. (2021). The tropical climatic region is located below 1000 m, with a hot summer and cold winter (Pradhan et al. 2013; Paudel et al. 2021); it is an important area of biodiversity in the country. However, in this region, only a few studies have documented butterflies covering part or all of the seasons of the year. For instance, Tamang et al. (2019) studied butterflies from pre‐monsoon to post‐monsoon, while Khanal (2006) covered only the post‐monsoon in the eastern lowlands. Khanal (2009) and Oli et al. (2023) studied butterflies from the western lowland districts. Moreover, the post‐monsoon and winter butterflies were studied in the Terai Annapurna Landscape (Suwal 2015). To our knowledge, there is no published literature on butterflies, nor their seasonality from the central lowland. Hence, the present research addresses this issue, studying butterflies across all the seasons of a year in the Institute of Forestry (IOF) Complex (a university campus), located in the central, tropical lowland of Nepal. The IOF complex consists of a mixture of natural forest and anthropogenic habitats (such as buildings, plantations, nurseries, and gardens), which buffer the dynamics of butterflies with seasons (Lourenço et al. 2020), and may yield different results than natural tropical habitats. Thus, this study may have a broad application to inform the butterfly seasonality in human‐interfered tropical landscapes.

Based on the prior knowledge of the role of seasonality on insect diversity and their populations (Kunte 1997; Silva et al. 2011; Habel et al. 2018), we also hypothesized that forest butterflies show seasonal variations in diversity and community composition in tropical habitats interfered with by human activities. We assume that wetter seasons accompany more overall species richness, abundance, and diversity, which also vary across taxonomic (family) levels. Hence, this study aimed to evaluate (a) the family‐level variation of butterfly diversity and (b) the seasonality of overall and family‐level diversity and community composition. Additionally, we also assessed the effect of precipitation (rainfall) and temperature on species richness and abundance. This study provides crucial information on butterflies' seasonal and climatic responses, useful for their conservation in tropical regions.

## 2 Materials and Methods

### 2.1 Study Area

**FIGURE 1:**
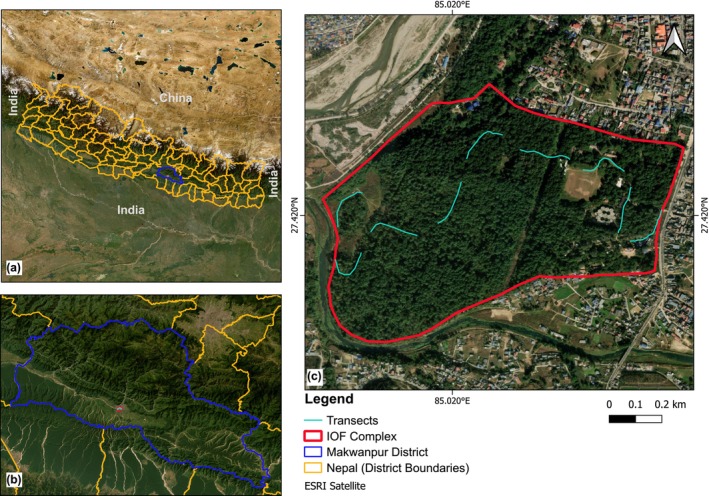
Map of the study area showing (a) District boundaries of Nepal, (b) Makwanpur District, and (c) Boundary of the IOF Complex, Hetauda (red polygon) and transects (bright teal lines). The map was prepared using QGIS version 3.36.2‐Maidenhead.

**FIGURE 2:**
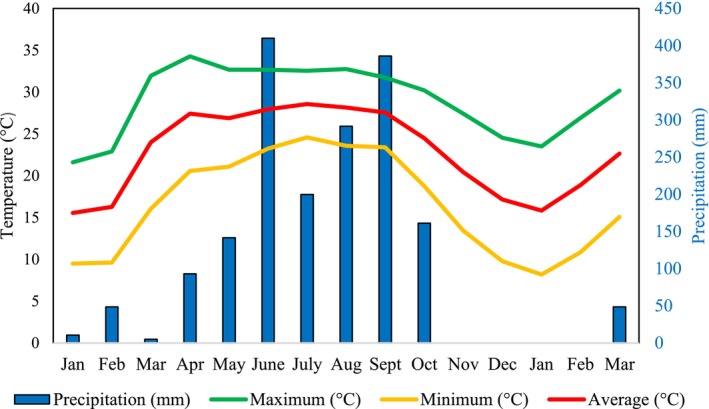
Bar and line plots showing monthly precipitation and temperature of Hetauda for 2022 (Jan–Dec) and 2023 (Jan–Mar). Months are on the X‐axis, temperature (°C) on the left Y‐axis, and precipitation (mm) on the right Y‐axis. The green, red, and orange lines indicate the maximum, average, and minimum temperatures, respectively.

The study was conducted in the Institute of Forestry (IOF) Complex, Hetauda, Makwanpur District, Nepal (Figure 1). The IOF Complex is located between the latitude of 27°25′16″ N and longitude of 85°01′27″ E, at an elevation range of 433–450 m above sea level, covering an area of 97 ha (0.97 km^2^) (Bajagain et al. 2020). Out of the total area, the forest constitutes 75.2 ha, followed by 10.6 ha of grassland patches, and the rest of the region belongs to campus buildings, nurseries, and open playgrounds (Bajagain et al. 2020). It serves as a heterogeneous habitat for different taxa, surrounded by the East–West (Mahendra) Highway in the east, the Rapti River in the west, the Karra River in the south, and settlements in the north. It lies in the lower tropical zone, having a warm temperate climate with an average annual temperature of 22.7°C and precipitation of 2,474 mm. Monsoon receives more rainfall than winter (Figure 2). The study area is characterized by lower tropical vegetation dominated by *Shorea robusta* (Dipterocarpaceae) forest. Grasses such as *Imperata cylindrica* and *Saccharum spontaneum* are prevalent in the grassland area. While *S. robusta, Bombax ceiba, Albizia lebbeck, Trewia nudiflora*, and planted *Eucalyptus* sp. are common in the remaining areas (Pandey et al. 2021). It provides the refugia for rich biodiversity, including more than 150 species of flora (Singh 2016), 132 species of birds (Bajagain et al. 2020), two species of turtle (Luitel et al. 2021), four species of mammals, and 11 species of snakes (Pradhan et al. 2020). The IOF Complex supports the habitat for a sound population of the *Axis axis* (Chital) (Shrestha and Dhami 2023).

### 2.2 Data Collection

Butterflies were sampled using the Pollard Walk survey (Pollard 1977) and checklist methods (Royer et al. 1998). Pollard Walk is more practical for long‐term monitoring of the species, while the checklist method is more efficient for the initial determination of the species list (Royer et al. 1998). It is possible that some species, especially those occupying the forest canopy and crown layer, may have been missing during the survey. According to the Pollard Walk (modified the length of transects), nine continuous fixed transects of 200 m each were laid, totaling 1,800 m, separated by ~100 m. The positions of transects were determined randomly based on accessibility for regular monitoring, covering all habitat types (buildings, open ground, forest, and grassland) in the study area. The transects are shown in Figure 1, which can be used for butterfly monitoring in the future. Butterflies were sampled for a year, from January to December 2022, covering all four seasons. Based on the rainfall, the seasons in Nepal are assigned as pre‐monsoon (Mar‐May), monsoon (June–Sept), post‐monsoon (Oct–Nov), and winter (Dec–Feb). Each transect was surveyed once a month, with a total replication of 12 for each, covering all transects on the same day to avoid the possibility of double‐counting the same individuals the next day. Butterflies were observed along the imaginary box of 5 m × 5 m × 5 m, at a constant pace, continuing to the transect: 2.5 m on either side of the transect, 5 m above, and 5 m ahead. Species' names and their numbers were documented. The checklist method was implemented 15 days after the transect survey each month. For this, species beyond the transects were randomly explored. The presence or absence of species was noted on the preliminary checklist, which contained hypothetically common species found in Nepal, and was created based on Smith (2011). Both types of sampling were done from 10:00 to 15:00 on sunny days to ensure the maximum detection of butterflies. The species were identified in the field using the butterfly guidebooks (Smith 2011; Smith et al. 2016). Species challenging to identify in the field were noted as unknown, including their count and morphological characters such as color pattern, approximate body size, and ocelli pattern. Besides, photographs of all different kinds of butterfly species were taken during field surveys with a Nikon D7000, Nikon AF‐S 55–250 mm, and smartphones. Most of them were photographed without being physically captured, although some were caught using a butterfly net and released. Species not recognized in the field surveys were later identified through internet sources, published literature (Tamang et al. 2019; Neupane and Miya 2021; Van der Poel and Smetacek 2022), as well as expert consultation. The IUCN status of butterflies was accessed from the IUCN Red List of Threatened Species (IUCN 2025).

The monthly precipitation (rainfall) and temperature data of Hetauda N.F.I. for 2022 (Jan–Dec) and 2023 (Jan–Mar) (Figure 2) were obtained from the Department of Hydrology and Meteorology, Kathmandu, Nepal. Hetauda N.F.I. (27°25′12.77″ N and 85°10′30.75″ E) is within the IOF Complex.

### 2.3 Data Analysis

The data obtained was analyzed in RStudio 4.4.2 using different packages and functions. The data from the Pollard Walk was used for the analysis of Hill diversity, Pielou's Evenness Index (J), nonmetric multidimensional scaling (NMDS), similarity percentage (SIMPER), and indicator species (IndVal). We computed Hill number (q), using “iNEXT” package, with 200 bootstrap replications and 95% confidence intervals (CIs) (Hill 1973; Hsieh et al. 2016; Chao et al. 2020) to compare the family‐level and overall seasonal butterfly diversity. Hill number uses the rarefaction and extrapolation approach to assess the diversity based on the reference sample. We estimated three forms of Hill diversity: species richness (q = 0), Hill Shannon diversity (q = 1, exponential of Shannon entropy or exponential Shannon Index), and Hill Simpson diversity (q = 2, inverse of Simpson concentration or inverse Simpson Index), using individual‐based abundance data (Chao et al. 2014). The Hill number (effective number of species) is statistically more rigorous than other diversity indices and integrates species richness and relative abundance (Chao et al. 2014; Roswell et al. 2021). Species richness emphasizes the presence or absence of species without considering their abundance, counting species equally. Hill Shannon diversity (hereafter exponential Shannon Index) is estimated based on the proportional count of species abundance, which can be interpreted as the effective number of common species in the given community. Hill Simpson diversity (hereafter inverse Simpson Index) is calculated based on the dominant species counts and denotes the effective number of dominant species in the given community (Chao et al. 2019; Roswell et al. 2021). Larger values of ‘q’ indicate greater richness and diversity in the community. There is no significant difference between the diversity indices of the groups compared when their CIs overlap. Pielou's Evenness Index (J) was calculated for each family and season to assess how uniformly individuals were distributed among the community, using the “vegan” package (Dixon 2003). Besides, to assess the differences in butterfly community composition between seasons, NMDS based on Bray–Curtis distance and analysis of similarities (ANOSIM) was analyzed using the “vegan” package (Dixon 2003). Likewise, species contribution to seasonal differences in community composition was assessed using similarity percentage analysis (SIMPER) based on Bray–Curtis dissimilarities, with ‘simper()’ function. Further, indicator species analysis (IndVal) was conducted to identify species significantly associated with specific seasons, using the “indicspecies” package, with 999 permutations (Cáceres and Legendre 2009). The indicator value ranges from 0 to 1, with 1 being a strong indicator of a particular season. The “ggplot2” and “ggthemes” packages were used for the data visualization from all the analyses (Wilkinson 2011; Arnold 2024). For the data obtained from the checklist method, only species richness (total number of species) was used to compare between seasons or families. The overall species richness for months/seasons and families was obtained from the sum of species recorded by both methods.

Generalized linear mixed models (GLMMs) were used to assess the effect of monthly precipitation and temperature (average) on species richness (overall, sum from both methods) and abundance (Pollard Walk). Precipitation and temperature were predictors, species richness and abundance were response variables, and family or month was a random factor (Bolker et al. 2009). Predictors were standardized to a mean of 0 and a standard deviation of 1. Poisson regression for richness and zero‐inflated negative binomial (ZINB) regression for the abundance model (due to overdispersion) were employed for this analysis using packages “lme4” and “glmmTMB” (Bates et al. 2024; Brooks et al. 2024). The Akaike Information Criterion (AIC) was calculated to select the best model, and model fit was assessed using the “DHARMa” package (Hartig 2024). CorelDRAW 9 was used to create image plates of butterflies.

## 3 Results

A total of 115 species of butterflies were documented during the study period from the IOF Complex. A complete list of butterflies with their scientific name, common name, abundance (*N*), and IUCN status is shown in Appendix A. The photographs of the butterflies are shown in Images 1–115 (Appendix D). Seventy‐eight species with 963 individuals were recorded from the Pollard Walk, and 91 species were documented from the checklist method, where 54 species were common to both methods. Twenty‐four species were unique to the Pollard Walk, while 37 were unique to the checklist method. Of the total species recorded, 19 are under the Least Concern (LC) category of IUCN, while the remaining are not evaluated (Appendix A).

### 3.1 Family‐Wise Variation of Butterfly Diversity

**FIGURE 3:**
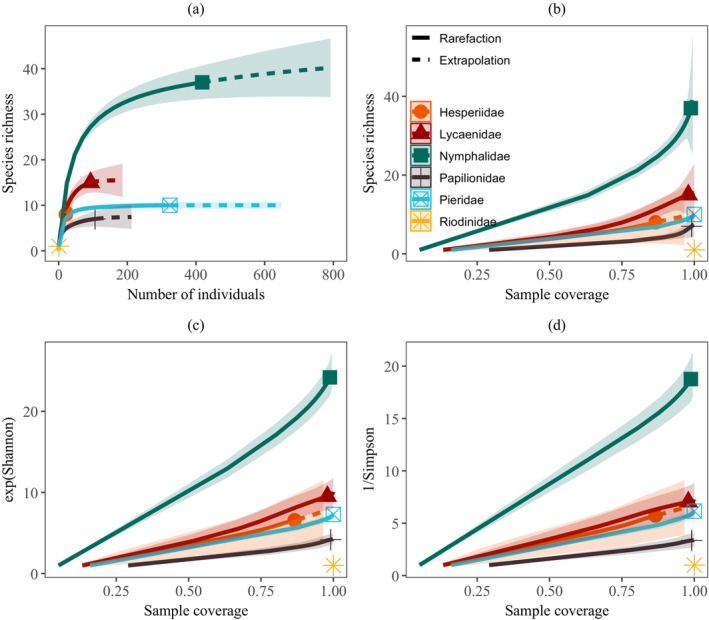
Rarefaction and extrapolation curves for butterfly species richness and diversity across different families. (a) Species accumulation curve based on the number of individuals, and (b) species richness, (c) exponential Shannon Index, and (d) inverse Simpson Index based on sample coverage. The solid curves represent rarefaction, the dashed curves represent extrapolation, and the shaded area denotes the corresponding 95% confidence intervals. There is no statistically significant difference in the diversity Index between the families when the CIs overlap.

The family Nymphalidae has the highest observed species richness (37 ± 9.1), followed by Lycaenidae (15 ± 3.7), and Riodinidae has the lowest richness (1.0 ± 0.0). There was a significant difference in species richness between butterfly families. The exponential Shannon and inverse Simpson diversities were significantly higher for Nymphalidae (24.2 ± 1.0 and 18.7 ± 0.9, respectively) and were significantly different from the rest of the families. There were no significant differences in diversity among the Pieridae, Lycaenidae, and Hesperiidae (Table 1, Figure 3). The overall richness was also highest for Nymphalidae (*S* = 48), followed by Lycaenidae (*S* = 27) when combining species from both methods. Nymphalidae also has the highest abundance (*N* = 418), followed by Pieridae (*N* = 324). Hesperiidae exhibited the highest evenness (0.91), followed by Nymphalidae (0.88) (Table 1).

**TABLE 1:** Hill diversity (q, Mean ± SE), abundance (*N*), sample coverage (SC), evenness (J), checklist richness, and overall richness across the butterfly families.

Families	Pollard walk						Checklist richness	Overall richness
	q = 0	q = 1	q = 2	N	SC	J		
Hesperiidae	8 ± 3.8	6.6 ± 1.7	5.7 ± 1.6	21	0.86	0.91	8	14
Lycaenidae	15 ± 3.7	9.5 ± 0.9	7.0 ± 0.9	93	0.97	0.83	22	27
Nymphalidae	37 ± 9.1*	24.2 ± 1.0*	18.7 ± 0.9*	418	0.98	0.88	40	48
Papilionidae	7 ± 1.5	4.2 ± 0.3	3.3 ± 0.3*	106	0.99	0.73	7	9
Pieridae	10 ± 0.3	7.3 ± 0.3	6.1 ± 0.3	324	1.00	0.86	14	16
Riodinidae	1 ± 0.0*	1.0 ± 0.0*	1.0 ± 0.0*	1	1.00	—	0	1

*Note:* The families with significant differences in ‘q’ and not overlapping CIs at *p* < 0.05 are indicated with asterisks '*'.

### 3.2 Seasonal Variation of Overall Butterfly Diversity and Community Composition

**FIGURE 4:**
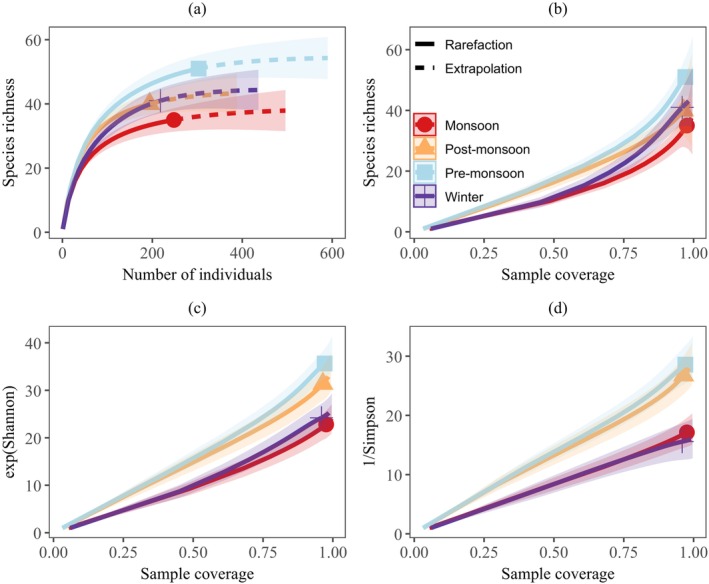
Rarefaction and extrapolation curves for butterfly species richness and diversity across different seasons. (a) Species accumulation curve based on the number of individuals, and (b) species richness, (c) exponential Shannon Index, and (d) inverse Simpson Index based on sample coverage. The solid curves represent rarefaction, the dashed curves represent extrapolation, and the shaded area denotes the corresponding 95% confidence intervals. There is no statistically significant difference in the diversity Index between the seasons when the CIs overlap.

The observed species richness significantly differed between pre‐monsoon and monsoon, but not between other seasons. It was significantly higher in pre‐monsoon (51 ± 5.3) than in monsoon (35 ± 6.4). The exponential Shannon and inverse Simpson diversities were significantly higher in pre‐monsoon (35.6 ± 1.7 and 28.6 ± 1.9, respectively) and post‐monsoon (31.4 ± 1.9 and 26.8 ± 2.2, respectively) than in other seasons. There was no significant difference in these diversities between pre‐monsoon and post‐monsoon, and winter and monsoon. Meanwhile, the diversity varied between pre‐monsoon/post‐monsoon and winter/monsoon (Table 2, Figure 4). The abundance was highest in pre‐monsoon (*N* = 303), followed by monsoon (*N* = 248), and lowest in post‐monsoon (*N* = 194). The evenness was highest in the post‐monsoon (0.93), followed by pre‐monsoon (0.91), monsoon (0.88), and lowest during winter (0.86) (Table 2). When combining the species from both methods, the highest species richness (*S* = 71) was reported during pre‐monsoon, and the lowest was in winter (*S* = 55) (Table 2). Overall, 20 species were reported in all four seasons.

**TABLE 2:** Hill diversity (q, Mean ± SE), abundance (*N*), sample coverage (SC), evenness (J), checklist richness, and overall richness of butterflies across the seasons.

Seasons	Pollard walk						Checklist richness	Overall richness
	q = 0	q = 1	q = 2	N	SC	J		
Pre‐monsoon	51 ± 5.3*	35.6 ± 1.7*	28.6 ± 1.9*	303	0.97	0.91	46	71
Monsoon	35 ± 6.4*	22.8 ± 1.5*	17.1 ± 1.5*	248	0.97	0.88	43	58
Post‐monsoon	40 ± 13.1	31.4 ± 1.9**	26.8 ± 2.2**	194	0.96	0.93	32	63
Winter	41 ± 7.8	24.2 ± 1.7**	15.6 ± 1.8**	218	0.95	0.86	34	55

*Note:* The seasons with significant differences in ‘q’ and not overlapping CIs at *p* < 0.05 are indicated with asterisks '*'.

**FIGURE 5:**
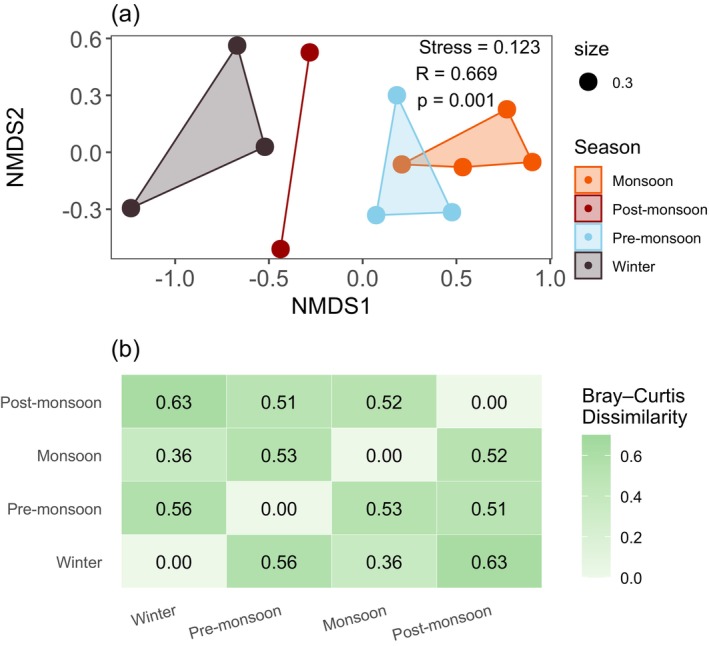
(a) Nonmetric multidimensional scaling (NMDS) ordination of all sampling units and (b) Bray–Curtis dissimilarity heatmap, indicating the relative differences in butterfly community composition between seasons (*p* < 0.05).

**FIGURE 6:**
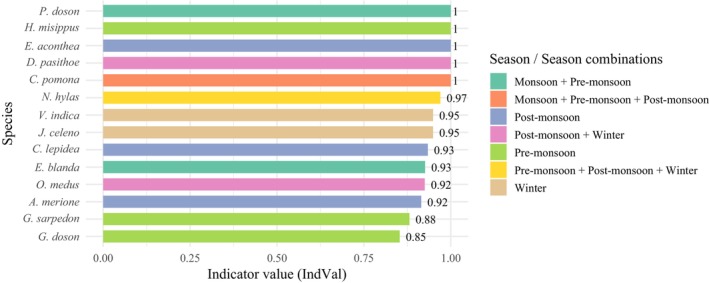
Bar plot showing the significant indicator species associated with a season or combinations of seasons (*p* < 0.05).

The NMDS ordination also showed a significant difference in the butterfly community composition between seasons, although there was a partial similarity between pre‐monsoon and monsoon (stress = 0.123 and *p* = 0.001) (Figure 5a). The greatest compositional dissimilarity was found between winter and post‐monsoon (Bray–Curtis = 0.63), and winter and pre‐monsoon (Bray–Curtis = 0.56) (Figure 5b). The SIMPER analysis identified species such as *Catopsilia pomona, Euthalia aconthea*, *Jamides celeno, Neptis hylas*, and *Pieris canidia* that emerged as top contributors to dissimilarity across seasons (Appendix B). A total of 14 species were identified as significantly associated with individual or combinations of seasons. *Euthalia aconthea* (IndVal = 1) and *Hypolimnas misippus* (IndVal = 1) were found to be the strong indicator species of post‐monsoon and pre‐monsoon, while *J. celeno* and *Vanessa indica* (IndVal = 0.949) were found to be strong indicators of winter. Other species were indicators for one or a combination of seasons (Figure 6 and Appendix C).

### 3.3 Seasonal Variation of Family‐Wise Butterfly Species Richness and Abundance

**FIGURE 7:**
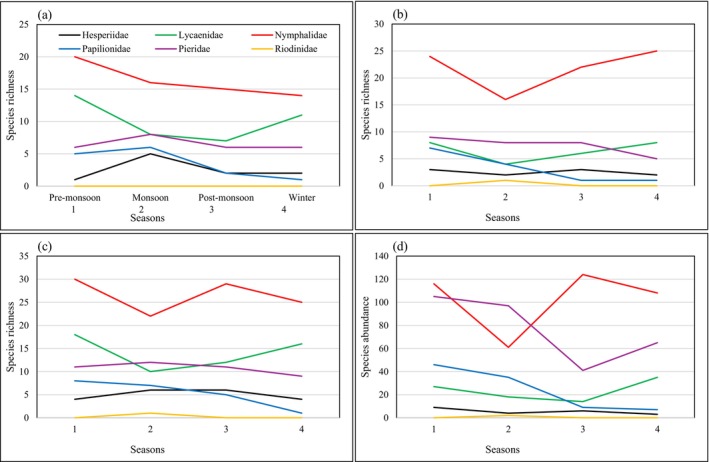
Line plots showing family‐wise butterfly species richness and abundance along seasons and methods: (a) Checklist (richness), (b) Pollard Walk (richness), (c) overall richness, and (d) abundance.

When combining the species from both methods, the family Hesperiidae showed the highest species richness during monsoon and post‐monsoon (*S* = 6) and the lowest during winter and pre‐monsoon (*S* = 4). Abundance was highest during pre‐monsoon (*N* = 9) and lowest during winter (*N* = 3) for this family. Lycaenidae and Nymphalidae have the highest species richness during pre‐monsoon (*S* = 18 and 30, respectively) and the lowest during monsoon (*S* = 10 and 22, respectively). Meanwhile, Nymphalidae has the highest abundance in post‐monsoon (*N* = 124) and the lowest during monsoon (*N* = 61). Lycaenidae has the highest abundance during winter (*N* = 35) and the lowest during post‐monsoon (*N* = 14). Papilionidae has the highest richness and abundance during pre‐monsoon (*S* = 8 and *N* = 46) and the lowest during winter (*S* = 1 and *N* = 7). Pieridae were relatively stable throughout the year, with species richness (monsoon = 12, pre‐monsoon and post‐monsoon = 11, and winter = 9), while the abundance was highest during pre‐monsoon (*N* = 105) and lowest during post‐monsoon (*N* = 41). One species of Riodinidae was recorded during the monsoon (Figure 7).

### 3.4 Effect of Monthly Precipitation and Temperature on Butterfly Species Richness and Abundance

**FIGURE 8:**
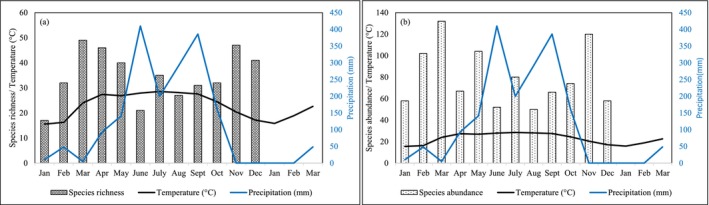
Bar and line plots showing (a) overall butterfly species richness and (b) abundance, with respect to months, monthly temperature (average), and precipitation of the study period. Months on the X‐axis, the black line indicates the monthly average temperature (°C) on the left Y‐axis, and the blue line indicates the monthly precipitation (mm) on the right Y‐axis.

**FIGURE 9:**
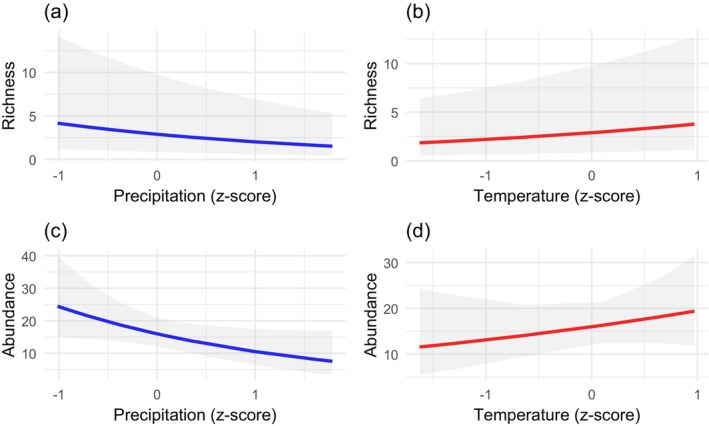
Line plots showing the effects of standardized (z‐score) monthly precipitation and standardized (z‐score) monthly average temperature on overall species richness and abundance based on GLLMs. The x‐axes represent the standardized predictors (mean = 0, standard deviation = 1). The y‐axes represent the predicted species richness (number of species) in (a) and (b) and predicted species abundance (number of individuals) in (c) and (d). Shaded areas represent the 95% confidence intervals around the predicted values.

The overall species richness and abundance were highest in March (*S* = 49 and *N* = 132), followed by November (*S* = 47 and *N* = 120). Richness was lowest in January (*S* = 17), and abundance was lowest in August (*N* = 50) (Figure 8). The GLMM analysis showed that monthly precipitation has a statistically significant negative effect on species richness (*p* < 0.05) and species abundance (*p* = 0.048). Meanwhile, the monthly average temperature has a statistically significant positive effect on richness (*p* < 0.05) but no significant effect on abundance (*p* = 0.355) (Table 3, Figure 9).

**TABLE 3:** Summary statistics of GLLMs showing the effect of monthly precipitation and average temperature on butterfly species richness and abundance.

Model	Effect	Estimate	Std. error	*z*	*p*
Richness (Poisson)	Intercept	1.06017	0.62346	1.7	0.089
	Temperature	0.27466	0.07544	3.641	0.0003*
	Precipitation	−0.36016	0.08071	−4.462	8.11E‐06*
Abundance (ZINB)	Intercept (Count part)	2.7713	0.1405	19.721	< 2e‐16
	Temperature (Count part)	0.1989	0.215	0.925	0.355
	Precipitation (Count part)	−0.42	0.2124	−1.977	0.048*
	Intercept (Zero‐inflation part)	−1.2514	0.3465	−3.612	0.0003

*Note:* Significant codes: 0 ‘***’ 0.001 ‘**’ 0.01 ‘*’ 0.05 ‘.’ 0.1 ‘’ 1.

## 4 Discussion

The IOF Complex, Hetauda, provides a home to 115 species of butterflies, which represent 16.54% of the total butterfly species recorded in Nepal (KC et al. 2025). Of the total species documented in our study, 16.52% fall under the Least Concern (LC) category of IUCN (IUCN 2025). Likewise, some butterfly species such as *Danaus chrysippus, Euploea mulcibe, Lampides boeticus*, and *Tirumala septentrionis* are known migratory butterflies (Chowdhury et al. 2021; Van der Poel and Smetacek 2022) and were found in the study area. Those butterflies may have been detected when they were using the resources there temporarily.

### 4.1 Family‐Wise Variation of Butterfly Diversity

The species richness, evenness, and abundance varied significantly among the families. The Hill Shannon and Simpson diversities significantly differed between Nymphalidae and the rest of the families. The distribution of individuals within the Hesperiidae and Nymphalidae was more balanced, shown by relatively high evenness, while the lower evenness in Papilionidae indicates dominance by one or a few species. Nymphalidae has the highest species richness, abundance, diversity, and dominance. The reason behind this finding could be attributed to the family being one of the most diverse groups of butterflies, with more than 6000 species distributed worldwide (van Nieukerken et al. 2011; Peña and Espeland 2015). Nepal alone hosts 239 species of Nymphalids (34.38% of the total species) (Van der Poel and Smetacek 2022; KC et al. 2025). Nymphalids are generalists, with more diverse host plants and a wide geographic range (Slove and Janz 2011; Nylin et al. 2014), characterized by large wingspan, active flight, and higher dispersal ability (Marini‐Filho and Martins 2010; Freire et al. 2021). Likewise, Nymphalids such as *E. malelas, N. hylas*, and *M. perseus* were present in large numbers and were widely distributed throughout the study area, occupying various habitats, which may have contributed to their higher abundance, evenness, diversity, and dominance. Similarly, Hesperiidae includes the species that are fast‐flying and commonly found in understory habitats, allowing them to be more uniformly distributed across the study area.

Our findings align with the previous studies from other regions of the tropical climate of Nepal. For example, Nymphalidae was reported as the species‐richest family, followed by Lycaenidae in the eastern lowlands (Subba and Tumbahangfe 2015; Tamang et al. 2019), eastern Siwalik (Bhusal and Khanal 2009), and the western lowlands (Khanal 2009; Sharma and Paudel 2021). Similar findings were also reported from the subtropical regions (Nepali et al. 2018; Shrestha et al. 2018; Khanal 2020; Miya et al. 2021; Neupane and Miya 2021; Subedi et al. 2021) and the temperate regions of Nepal (Prajapati et al. 2000; Shrestha 2016; Shrestha et al. 2020). Studies in tropical dry climatic regions of India have also found results similar to our findings (Tiple et al. 2007; Tiple and Khurad 2009; Boruah et al. 2018). From previous and present findings, it can be inferred that the generalist butterfly families are widely adapted to various climatic regions in their dominant form.

### 4.2 Seasonal Variation of Overall Butterfly Diversity and Community Composition

The observed species richness varied significantly between the pre‐monsoon and monsoon. The pre‐monsoon has considerably higher richness, exponential Shannon diversity, and inverse Simpson diversity, followed by the post‐monsoon. The diversities significantly varied between pre‐monsoon/post‐monsoon and winter/monsoon. Likewise, overall species richness showed two seasonal peaks: pre‐monsoon and post‐monsoon. However, the abundance was observed to be highest in pre‐monsoon, followed by monsoon, and lowest in post‐monsoon. The species were most evenly distributed in the post‐monsoon, whereas winter showed a slightly more uneven distribution. 17.39% of the recorded species were observed in all four seasons. The butterfly community composition varied significantly between the seasons, with a partial similarity between pre‐monsoon and monsoon, and the greatest dissimilarity between winter and post‐monsoon. Species such as *C. pomona, E. aconthea*, *J. celeno, N. hylas*, and *P. canidia* contributed most to the dissimilarity across seasons. Some species like *E. aconthea*, *H. misippus* , *J. celeno*, and *V. indica* were found as the indicator species and were significantly associated with individual or combinations of seasons. The seasonality of butterflies may be attributed to climatic parameters that change the butterflies' habitat conditions. The increased species richness, abundance, and diversity during the pre‐monsoon could be due to the higher abundance of larvae in the dry season, as they are relatively free from predators. Their emergence coincides with the onset of the rainy season, providing favorable conditions for flight and oviposition due to the presence of younger leaves compared to other seasons (Morais et al. 1999; Júnior and Diniz 2015). The first rain can potentially induce increased insect activity (Wolda 1988). Dry and wet seasons are the primary factors influencing butterfly diversity and seasonality (Meléndez‐Jaramillo et al. 2019; Shuey et al. 2024). The convergence of seasonal peaks for numerous abundant butterfly species (or families) may result in higher abundance during specific months or seasons (Gupta et al. 2019). The biannual peak in richness and abundance indicates the succession of generations, achievable only if the species can withstand more significant climatic variability and utilize local resources more efficiently over extended periods (Wolda 1978). Besides, the host plant availability could explain the seasonal pattern in butterfly abundance and species richness (Valtonen et al. 2013).

Our findings are in line with a previous study from the tropical region in eastern Siwalik (Bhusal and Khanal 2009), subtropical regions (Nepali et al. 2018; Miya et al. 2021), and a temperate region (Prajapati et al. 2000). While the findings contrast with other tropical and subtropical studies that reported higher species richness, abundance, or diversity in post‐monsoon (Rai 2017; Oli and Sharma 2019; Chaudhary 2023; Oli et al. 2023), monsoon (Thapa 2008; Tamang et al. 2019; Neupane and Miya 2021), and winter (Sah 2019). Likewise, two seasonal peaks of butterfly richness and abundance in late monsoon and early winter, as well as pre‐monsoon and post‐monsoon, were also observed in India and Bhutan (Kunte 1997; Singh 2012; Naik et al. 2022). The contrasting seasonal variation in different regions may be due to differences in geography (elevation) and other bioclimatic factors, such as vegetation, affecting the butterfly species composition (Cómbita et al. 2022).

The butterfly community composition across the season contrasts with the findings in the subtropical lowland of Bhutan, where the greatest similarity was found between post‐monsoon and pre‐monsoon, and the lowest similarity was between winter and pre‐monsoon/monsoon (Singh 2012). Likewise, higher similarity in community composition was observed between the early and late rainy seasons in the semi‐warm subhumid climate of Mexico (Meléndez‐Jaramillo et al. 2019). Furthermore, species composition was found to be significantly similar between the rainy and winter seasons in Telangana, Jammu, and Kashmir (Sharma and Sharma 2021; Ravivarma et al. 2023), while the slightest similarity was noted between pre‐monsoon (Mar‐May) and post‐monsoon (Oct‐Nov) in the tropical Northeast India (Singh et al. 2015).

### 4.3 Seasonal Variation of Family‐Wise Species Richness and Abundance

The Hesperiidae family exhibited the greatest species richness during the monsoon/post‐monsoon and the lowest in winter/pre‐monsoon. The Lycaenidae and Nymphalidae demonstrated the highest richness in the pre‐monsoon and the lowest during the monsoon. The Papilionidae showed the greatest richness in the pre‐monsoon and the least in winter. The Lycaenidae were most abundant in winter, while the Hesperiidae, Nymphalidae, and Papilionidae had their highest abundance in the pre‐monsoon. The Pieridae remained relatively stable across all seasons, with the highest abundance occurring in the pre‐monsoon and the lowest in the post‐monsoon. The Riodinidae were recorded only in the monsoon. These findings provided valuable insights into the responses of the butterfly families to seasonal changes in the tropical region. The onset of the monsoon creates the availability of mud‐puddling sites and nectar resources suitable for generalist species of Nymphalidae, Lycaenidae, and Pieridae, peaking their diversity and abundance (Mahata et al. 2024). Specialist species concentrate during the post‐monsoon season as monsoon supports a significant number of plant species and provides favorable growth of caterpillars (Kunte 1997; Harrison et al. 2020).

No specific studies previously explained the seasonal variations of butterfly families from Nepal. In Tamil Nadu, India, the abundance of Nymphalidae remained consistent across all seasons except summer, while Pieridae peaked in post‐monsoon, and Lycaenidae were most abundant during winter (Hussain et al. 2011). In the *S. robusta* forest of Jharkhand, Nymphalidae and Lycaenidae showed maximum richness during the rainy season and a decrease in summer, whereas Pieridae and Papilionidae showed only slight seasonal variations (Verma 2009). Likewise, in Telangana, Papilionidae and Pieridae showed the highest occurrence in winter, with similar occurrence between rainy and winter for Lycaenidae, Nymphalidae, and Hesperiidae (Ravivarma et al. 2023). Similarly, the study in the tropical region of Mexico found the greatest species richness and abundance of Lycaenidae, Nymphalidae, and Pieridae in the rainy season (June‐Sept), while the maximum richness of Papilionidae was in the dry season (Feb‐May) (Pozo et al. 2008). In the tropical regions of Brazil, Nymphalidae were found to be concentrated more between post‐monsoon and pre‐monsoon (Freire et al. 2023; Ribeiro et al. 2010). Whereas Nymphalidae abundance peaked in the wet season and decreased in the dry season in the neotropical and semiarid regions (Nobre et al. 2012; Freitas et al. 2021). The transition between the wet and dry seasons (Sept‐Nov) represented the highest species richness and abundance of Nymphalidae in the Cerrado Biome and Rio Doce State Park (Júnior and Diniz 2015; Lourenço et al. 2020). However, another study found that the Nymphalidae did not vary between dry and wet seasons in the eastern extreme of the Amazon region and the mountaintop archipelago (Pereira et al. 2017; Araujo et al. 2020). Lycaenidae, Riodinidae, and Hesperiidae increased during the rainy season in Belize (Shuey et al. 2024). Conversely, Hesperiidae were richest and most abundant during the dry season (humid) in San José, Costa Rica (Murillo‐Hiller et al. 2019). These findings suggest that species richness and abundance fluctuate across seasons differently for various butterfly families, which in turn vary by geography and bioclimatic regions.

### 4.4 Effect of Monthly Precipitation and Temperature on Overall Butterfly Species Richness and Abundance

The GLLM analysis showed that butterfly species richness and abundance tend to decrease with increased precipitation. An explanation could be that excessive precipitation might alter ecological conditions unfavorably for certain species and may cause flooding or wash away food sources, decreasing habitat quality. Meanwhile, species were positively associated with increased temperature. Temperature influences daily activities such as flight and foraging movement, while precipitation indirectly affects the availability of nectar and host resources (Gullan and Cranston 2014; Kumar et al. 2023). Moreover, temperature influences butterflies indirectly through rainfall, atmospheric pressure, wind, humidity, and the growth of vegetation (Khanal 2013). Warmer temperatures can create more favorable conditions for various species of generalist families (e.g., Nymphalidae) (Ribeiro and Freitas 2010).

Our findings are similar to some and contrasting to other studies from different regions of the world. Both temperature and precipitation were noted as crucial factors for butterfly population dynamics in Europe (Mills et al. 2017; Herrando et al. 2019). Temperature (negative correlation) better explained species occurrence than precipitation, while the opposite pattern was found for abundance in the Mediterranean climatic region of Israel (Comay et al. 2021). Rainfall was positively correlated, and the temperature was negatively correlated with butterfly species richness and abundance in coffee–banana agroforests in Uganda (Munyuli 2013). Monthly temperature and precipitation were significantly correlated with species richness in Belize (Shuey et al. 2024). In the subtropical Uttarakhand, India, a weak positive linear relationship was found between maximum temperature, rainfall, relative humidity (morning and evening), and species abundance (Samraj and Agnihotri 2021). Likewise, the richness and abundance were significantly correlated with the temperature in a subtropical habitat of Delhi (Gupta et al. 2019). Temperature and relative humidity were the most significant factors for butterfly richness in the tropical dry forest of the Eastern Ghats (Mahata et al. 2023). The abundance correlated with precipitation but not temperature in the tropical Amazonian Ecuador (Grøtan et al. 2012). Temperature, humidity, and vegetation structure were significant predictors of butterfly composition and abundance in the Neotropical dry forests of Western Ecuador (Checa et al. 2014). Ribeiro et al. (2010) found no significant association between rainfall and species richness/abundance in the Atlantic Forest of Brazil. Khanal et al. (2013) found the highest butterfly number at higher temperatures. The precipitation was correlated with species richness, while relative humidity was highly correlated with abundance in Mexico (Meléndez‐Jaramillo et al. 2019). In another study, in a Mountain Area of the Northern Iberian Peninsula, the species abundance was found to be related to larval food plants, suggesting the local abundance is influenced by local resources while regional distribution is limited by climatic tolerance of butterfly species (Gutiérrez and Menéndez 1995). Besides the two factors (temperature and precipitation), several other factors could better explain the availability of butterflies, such as body size, humidity, preferred food plants, altitude, and habitat types (Bhusal and Khanal 2009; Khanal et al. 2013; Pandey et al. 2017).

## 5 Conclusion

Despite its small area, the Institute of Forestry Complex, Hetauda, hosts 115 species of butterflies. The study highlights the significant fluctuations of butterfly diversity across seasons and families. With two seasonal peaks of diversity– pre‐monsoon and post‐monsoon—the precipitation negatively affected and temperature positively impacted the species richness and abundance. The butterfly community composition significantly varied between the seasons, with the contribution of multiple species. Species such as *E. aconthea*, *H. misippus* , *J. celeno*, and *V. indica* were found to be the indicators of certain seasons. Nymphalidae was the most diverse and abundant family, contributing to the overall diversity. The findings of this study are consistent with studies from different climatic regions of the world, where butterfly diversity tends to change with seasonality and shows two peaks. However, the negative effect of precipitation, which is crucial for host plant resource availability, contrasts with previous findings, suggesting other possible factors influencing the butterfly community in the study area. The present study included only two climatic variables; thus, incorporating additional factors like humidity, host plant availability, habitat types, and anthropogenic disturbances could better explain butterfly diversity variations in the study area. Moreover, the Pollard Walk survey and checklist methods may not have covered all the species in the area, especially those occupying tree canopies. Therefore, we recommend incorporating other methods, such as bait traps, for future studies.

## Author Contributions

**Mahamad Sayab Miya:** conceptualization (lead), data curation (lead), formal analysis (lead), methodology (lead), software (lead), supervision (lead), validation (lead), visualization (lead), writing – original draft (lead), writing – review and editing (equal). **Subarna Shrestha:** conceptualization (supporting), investigation (equal), methodology (supporting), writing – review and editing (equal). **Annapurna Dhakal:** investigation (equal), writing – review and editing (equal). **Soniya Shrestha:** investigation (equal), writing – original draft (supporting), writing – review and editing (equal). **Renuka Karki:** investigation (equal), writing – review and editing (equal). **Kriti Thapa:** investigation (equal), writing – review and editing (equal). **Nisha Simkhada:** investigation (equal), writing – review and editing (equal). **Prakriti Chataut:** investigation (equal), writing – original draft (supporting), writing – review and editing (equal). **Pabitra Maya Tamang:** investigation (equal), writing – review and editing (equal). **Apeksha Chhetri:** data curation (supporting), formal analysis (supporting), writing – review and editing (equal).

## Conflicts of Interest

The authors declare no conflicts of interest.

## Data Availability

All data is included in the paper's main text and appendices.

## Appendix A List of Butterflies Documented From the IOF Complex, Hetauda

**Table:**

S.N.	Scientific name	Common name	*N*	IUCN status
	**Family ‐ Hesperiidae**			
1	*Aeromachus pygmaeus* (Fabricius, 1775)	Pigmy Scrub Hopper**	2	NE
2	*Ancistroides nigrita* (Latreille, [1824])	Chocolate Demon**	5	NE
3	*Borbo cinnara* (Wallace, 1866)	Rice Swift	1	NE
4	*Iambrix salsala* (Moore, [1866])	Chestnut Bob*		NE
5	*Notocrypta curvifascia* (C. & R. Felder, 1862)	Restricted Demon*		NE
6	*Parnara guttatus* (Bremer & Grey, [1852])	Straight Swift*		NE
7	*Pelopidas agna* (Moore, [1866])	Obscure‐branded Swift*		NE
8	*Pelopidas sinensis* (Mabille, 1877)	Large‐branded Swift**	1	NE
9	*Pseudoborbo bevani* (Moore, 1878)	Bevan's Swift**	5	NE
10	*Sarangesa dasahara* (Moore, [1866])	Common Small Flat*		NE
11	*Tagiades gana* (Moore, [1866])	Suffused Snow Flat	4	NE
12	*Tagiades litigiosa* Möschler, 1878	Water Snow Flat**	1	NE
13	*Telicota bambusae* (Moore, 1878)	Dark Palm Dart**	2	NE
14	*Udaspes folus* (Cramer, [1775])	Grass Demon*		NE
	**Family ‐ Lycaenidae**			
15	*Acytolepis puspa* (Horsfield, [1828])	Common Hedge Blue*		NE
16	*Arhopala centaurus* (Fabricius, 1775)	Centaur Oakblue	7	NE
17	*Arhopala eumolphus* (Cramer, [1780])	Green Oakblue*	22	NE
18	*Castalius rosimon* (Fabricius, 1775)	Common Pierrot**	3	NE
19	*Catochrysops strabo* (Fabricius, 1793)	Forget‐me‐not	4	NE
20	*Cheritra freja* (Fabricius, 1793)	Common Imperial**	1	LC
21	*Chilades lajus* (Stoll, [1780])	Lime Blue	1	LC
22	*Chliaria othona* (Hewitson, 1865)	Orchid Tit*		NE
23	*Curetis acuta* Moore, 1877	Angled Sunbeam*		NE
24	*Euchrysops cnejus* (Fabricius, 1798)	Gram Blue*		NE
25	*Jamides celeno* (Cramer, [1775])	Common Cerulean	20	NE
26	*Lampides boeticus* (Linnaeus, 1767)	Peablue*		LC
27	*Leptotes plinius* (Fabricius, 1793)	Zebra Blue	4	NE
28	*Loxura atymnus (Stoll, 1780)*	Yamfly	5	NE
29	*Luthrodes pandava* (Horsfield, [1829])	Plains Cupid*		NE
30	*Megisba malaya* (Horsfield, [1828])	Malayan*		NE
31	*Nakaduba kurava* (Moore, [1858])	Transparent Six‐line Blue*		NE
32	*Poritia hewitsoni* Moore, [1866]	Common Gem*		NE
33	*Prosotas dubiosa* (Semper, [1879])	Tailless Lineblue*		NE
34	*Prosotas nora* (C. Felder, 1860)	Common Lineblue**	4	NE
35	*Pseudozizeeria maha* (Kollar, [1844])	Pale Grass Blue	14	NE
36	*Rapala manea* (Hewitson, 1863)	Slate Flash	2	NE
37	*Rapala pheretima* (Hewitson, 1863)	Copper Flash	2	NE
38	*Taraka hamada* (Druce, 1875)	Forest Pierrot**	2	NE
39	*Zeltus amasa* (Hewitson, 1865)	Fluffy Tit*		NE
40	*Zizeeria karsandra* (Moore, 1865)	Dark Grass Blue	2	LC
41	*Zizina otis* (Fabricius, 1787)	Lesser Grass Blue*		LC
	**Family ‐ Nymphalidae**			
42	*Acraea violae* (Fabricius, 1793)	Tawny Coster	1	NE
43	*Aglais caschmirensis* (Kollar, [1844])	Indian Tortoiseshell	11	NE
44	*Argynnis hyperbius* (Linnaeus, 1763)	Indian Fritillary**	2	NE
45	*Ariadne ariadne* (Linnaeus, 1763)	Angled Castor*		NE
46	*Ariadne merione* (Cramer, [1777])	Common Castor	16	NE
47	*Athyma nefte* (Cramer, [1780])	Color Sergeant**	1	NE
48	*Athyma perius* (Linnaeus, 1758)	Common Sergeant	5	NE
49	*Cethosia biblis* (Drury, [1773])	Red Lacewing*		NE
50	*Cupha erymanthis* (Drury, [1773])	Rustic*		NE
51	*Cynitia lepidea* (Butler, 1868)	Gray Count	10	NE
52	*Cyrestis thyodamas* Boisduval, 1846	Common Map*		NE
53	*Danaus chrysippus* (Linnaeus, 1758)	Plain Tiger	27	LC
54	*Danaus genutia* (Cramer, [1779])	Common Tiger	6	NE
55	*Discophora sondaica* Boisduval, [1836]	Common Duffer*		NE
56	*Elymnias hypermnestra* (Linnaeus, 1763)	Common Palmfly	3	NE
57	*Elymnias malelas* (Hewitson, 1863)	Spotted Palmfly*		NE
58	*Euploea core* (Cramer, [1780])	Common Indian Crow	42	LC
59	*Euploea mulciber* (Cramer, [1777])	Striped Blue Crow*		NE
60	*Euthalia aconthea* (Cramer, [1777])	Common Baron	12	NE
61	*Hypolimnas bolina* (Linnaeus, 1758)	Great Eggfly	3	NE
62	*Hypolimnas misippus* (Linnaeus, 1764)	Danaid Eggfly**	8	LC
63	*Junonia almana* (Linnaeus, 1758)	Peacock Pansy	11	LC
64	*Junonia atlites* (Linnaeus, 1763)	Gray Pansy	18	NE
65	*Junonia hierta* (Fabricius, 1798)	Yellow Pansy**	1	LC
66	*Junonia iphita* (Cramer, [1779])	Chocolate Pansy	20	NE
67	*Junonia lemonias* (Linnaeus, 1758)	Lemon Pansy	18	NE
68	*Junonia orithya* (Linnaeus, 1758)	Blue Pansy	1	LC
69	*Lethe confusa* Aurivillius, 1898	Banded Treebrown**	3	NE
70	*Melanitis leda* (Linnaeus, 1758)	Common Evening Brown	5	LC
71	*Melanitis phedima* (Cramer, [1780])	Dark Evening Brown	4	NE
72	*Mycalesis malsara* (Moore, 1858)	White‐line Bushbrown**	4	NE
73	*Mycalesis mineus* (Linnaeus, 1758)	Dark‐brand Bushbrown	3	NE
74	*Mycalesis perseus* (Fabricius, 1775)	Common Bushbrown	35	NE
75	*Mycalesis visala* Moore, [1858]	Long‐brand Bushbrown**	2	NE
76	*Neptis cartica* Moore, 1872	Plain Sailer*		NE
77	*Neptis hylas* (Linnaeus, 1758)	Common Sailer	45	NE
78	*Orsotriaena medus* (Fabricius, 1775)	Jungle Brown	24	NE
79	*Pantoporia hordonia* (Stoll, [1790])	Common Lascar**	3	NE
80	*Parantica aglea* (Stoll, [1782])	Glassy Tiger	18	NE
81	*Pseudergolis wedah* (Kollar, 1848)	Tabby*		NE
82	*Symbrenthia lilaea* (Hewitson, 1864)	Common Jester	11	NE
83	*Tanaecia julii* (Lesson, 1837)	Common Earl	7	NE
84	*Tirumala limniace* (Cramer, [1775])	Blue Tiger	1	NE
85	*Tirumala septentrionis* (Butler, 1874)	Dark Blue Tiger*		NE
86	*Vagrans egista* (Cramer, [1780])	Vagrant*		NE
87	*Vanessa indica* (Herbst, 1794)	Indian Red Admiral	10	NE
88	*Ypthima baldus* (Fabricius, 1775)	Common Five‐ring	15	NE
89	*Ypthima huebneri* Kirby, 1871	Common Four‐ring	12	NE
	**Family ‐ Papilionidae**			
90	*Graphium agamemnon* (Linnaeus, 1758)	Tailed Jay*		NE
91	*Graphium doson* (C. & R. Felder, 1864)	Common Jay	9	NE
92	*Graphium sarpedon* (Linnaeus, 1758)	Common Bluebottle	18	LC
93	*Pachliopta aristolochiae* (Fabricius, 1775)	Common Rose**	1	LC
94	*Papilio clytia* Linnaeus, 1758	Common Mime**	2	NE
95	*Papilio demoleus* Linnaeus, 1758	Lime Swallowtail	25	NE
96	*Papilio memnon* Linnaeus, 1758	Great Mormon*		NE
97	*Papilio nephelus* Boisduval, 1836	Yellow Helen	3	NE
98	*Papilio polytes* Linnaeus, 1758	Common Mormon	48	NE
	**Family ‐ Pieridae**			
99	*Belenois aurota* (Fabricius, 1793)	Pioneer*		LC
100	*Appias lyncida* (Cramer, [1777])	Chocolate Albatross*		NE
101	*Catopsilia pomona* (Fabricius, 1775)	Common Emigrant	61	NE
102	*Catopsilia pomona pomona* (Fabricius, 1775)	Lemon Emigrant	30	NE
103	*Catopsilia pyranthe* (Linnaeus, 1758)	Mottled Emigrant	28	NE
104	*Delias descombesi* (Boisduval, 1836)	Red‐spot Jezebel*		NE
105	*Delias hyparete* (Linnaeus, 1758)	Painted Jezebel	2	NE
106	*Delias pasithoe* (Linnaeus, 1767)	Red‐base Jezebel	9	NE
107	*Eurema blanda* (Boisduval, 1836)	Three‐spot Grass Yellow	19	NE
108	*Eurema brigitta* (Stoll, [1780])	Small Grass Yellow**	17	LC
109	*Eurema hecabe* (Linnaeus, 1758)	Common Grass Yellow	74	LC
110	*Ixias pyrene* (Linnaeus, 1764)	Yellow Orange Tip*		NE
111	*Leptosia nina* (Fabricius, 1793)	Psyche*		NE
112	*Pieris brassicae (Linnaeus, 1758)*	Large Cabbage White**	11	LC
113	*Pieris canidia* (Linnaeus, 1768)	Indian Cabbage White	73	NE
114	*Pontia daplidice* (Linnaeus, 1758)	Bath White*		LC
	**Family ‐ Riodinidae**			
115	*Abisara bifasciata* Moore, 1877	Double‐banded Judy**	1	NE

*Note:* *Species unique to the checklist method, **Species unique to the Pollard Walk, and the remaining are common to both methods. Abbreviation used: *N* = abundance, NE = Not Evaluated, LC = Least Concern.

## Appendix B Top Five Butterfly Species Contributing to Community Dissimilarity Between Seasonal Pairs Based on SIMPER Analysis

**Table:**

Species	Average dissimilarity	SD	Ratio	A avg.	B avg.	Cumulative (%)	*p*
**Winter vs. pre‐monsoon**							
*P. canidia*	0.05324	0.03814	1.396	12.667	5.333	7.70	0.117
*C. pomona*	0.04304	0.02442	1.763	0	7.333	13.90	**0.043**
*C. pomona pomona*	0.03369	0.02678	1.258	0	5.333	18.70	**0.044**
*J. celeno*	0.03115	0.01515	2.056	6	0.667	23.20	0.068
*G. sarpedon*	0.02798	0.01882	1.487	0	4.333	27.20	**0.016**
**Winter vs. monsoon**							
*P. canidia*	0.07045	0.04533	1.554	12.667	2.75	9.40	**0.007**
*C. pomona*	0.05605	0.01398	4.01	0	7.5	16.90	**0.002**
*J. celeno*	0.0444	0.01537	2.888	6	0	22.80	**0.001**
*E. hecabe*	0.03729	0.02534	1.472	6.333	6.75	27.80	0.057
*N. hylas*	0.03611	0.03163	1.142	5	1	32.60	**0.031**
**Winter vs. post‐monsoon**							
*P. canidia*	0.05187	0.03831	1.354	12.667	4	7.90	0.182
*E. aconthea*	0.03827	0.01979	1.934	0	6	13.70	**0.011**
*J. celeno*	0.03557	0.01365	2.605	6	0	19.10	**0.037**
*A. merione*	0.03302	0.01173	2.816	0.667	6	24.20	**0.012**
*C. pomona*	0.02785	0.00844	3.301	0	4.5	28.40	0.641
**Pre‐monsoon vs. monsoon**							
*C. pomona pomona*	0.02748	0.02254	1.219	5.333	2.25	5.20	0.171
*P. polytes*	0.0273	0.0224	1.219	3.333	5.5	10.30	0.339
*N. hylas*	0.02426	0.01295	1.874	4.667	1	14.90	0.505
*C. pomona*	0.02407	0.01242	1.938	7.333	7.5	19.50	0.911
*G. sarpedon*	0.02262	0.01897	1.192	4.333	1.25	23.70	**0.059**
**Pre‐monsoon vs. post‐monsoon**							
*E. aconthea*	0.03261	0.01671	1.951	0	6	5.00	**0.010**
*A. merione*	0.03198	0.0113	2.83	0	6	9.90	**0.010**
*O. medus*	0.02672	0.01076	2.483	0.667	6	14.00	**0.034**
*G. sarpedon*	0.02418	0.01637	1.477	4.333	0	17.70	0.115
*C. pomona pomona*	0.02363	0.02189	1.08	5.333	2.5	21.30	0.407
**Monsoon vs. post‐monsoon**							
*E. aconthea*	0.04067	0.02028	2.005	0	6	6.20	**0.001**
*A. merione*	0.03685	0.01521	2.423	0.5	6	11.80	**0.001**
*O. medus*	0.0322	0.01299	2.478	0.75	6	16.70	**0.001**
*N. hylas*	0.03158	0.01291	2.447	1	6	21.50	0.202
*C. lepidea*	0.02536	0.01183	2.144	0.25	4	25.40	**0.003**

*Note:* A and B refer to the average abundance of the species in the first and second seasons of each contrast, respectively. Cumulative contributions (%) and significance levels (*p*‐values from permutations) indicate the species most responsible for seasonal compositional changes. The significant *p*‐values are in bold (*p* < 0.05).

## Appendix C Indicator Species Analysis (IndVal) Results Showing Butterfly Species Significantly Associated With Seasonal Groups

**Table:**

Season/Combination	Species	Indicator value	*p*
Post‐monsoon	*E. aconthea*	1	0.018
	*C. lepidea*	0.934	0.028
	*A. merione*	0.915	0.02
Pre‐monsoon	*H. misippus*	1	0.014
	*G. sarpedon*	0.881	0.027
	*G. doson*	0.853	0.045
Winter	*J. celeno*	0.949	0.01
	*V. indica*	0.949	0.011
Monsoon + pre‐monsoon	*P. doson*	1	0.008
	*E. blanda*	0.926	0.038
Post‐monsoon + winter	*D. pasithoe*	1	0.008
	*O. medus*	0.925	0.032
Monsoon + post‐monsoon + pre‐monsoon	*C. pomona*	1	0.01
Post‐monsoon + pre‐monsoon + winter	*N. hylas*	0.97	0.013

*Note:* Only species with statistically significant associations (*p* < 0.05) are shown.

## Appendix D Images of Butterflies Documented From the IOF Complex, Hetauda

Images 1–20. 1—Pigmy Scrub Hopper | 2—Rice Swift | 3—Chocolate Demon | 4—Chestnut Bob | 5—Restricted Demon | 6—Straight Swift | 7—Obscure‐banded Swift | 8—Large‐branded Swift | 9—Bevan's Swift | 10—Common Small Flat | 11—Suffused Snow Flat | 12—Water Snow Flat | 13—Dark Palm Dart | 14—Grass Demon | 15—Common Hedge Blue | 16—Green Oakblue | 17—Centaur Oakblue | 18—Common Pierrot | 19—Forget‐me‐not | 20—Common Imperial. Photos: N. Pradhan (1, 3, and 8), A. Dhakal (2, 11, and 15), P. Chataut (4, 6, 7, 10, 14, 16, and 19), N. Simkhada (5), Soniya Shrestha (9 and 13), Subarna Shrestha (12, 17, and 20), and K. Thapa (18).

Images 21–40. 21—Lime Blue | 22—Orchid Tit | 23—Angled Sunbeam | 24—Gram Blue | 25—Common Cerulean | 26—Peablue | 27—Zebra Blue | 28—Yamfly | 29—Plains Cupid | 30—Malayan | 31—Transparent Six‐line Blue | 32—Common Gem | 33—Tailless Libeblue | 34—Common Lineblue | 35—Pale Grass Blue | 36—Slate Flash | 37—Copper Flash | 38—Forest Pierrot | 39—Fluffy Tit | 40—Dark Grass Blue. Photos: P. Chataut (21, 22, 23, 26, 29, 30, 31, 32, 34, 36, 37, 39, and 40), A. Dhakal (24), Subarna Shrestha (25, 27, 35, and 38), and N. Simkhada (28 and 33).

Images 41–60. 41—Lesser Grass Blue | 42—Tawny Coster | 43—Indian Tortoiseshell | 44—Indian Fritillary | 45—Common Castor (Unable to photograph Angled Castor) | 46—Common Castor | 47—Color Sergeant | 48—Common Sergeant | 49—Red Lacewing | 50—Rustic | 51—Gray Count | 52—Common Map | 53—Plain Tiger | 54—Common Tiger | 55—Common Duffer | 56—Common Palmfly | 57—Spotted Palmfly | 58—Common Indian Crow | 59—Striped Blue Crow | 60—Common Baron. Photos: P. Chataut (41, 42, 43, 45, 48, 51, 52, and 55), Subarna Shrestha (44, 46, 54, 56, and 58), M.S. Miya (47 and 60), P.M. Tamang (49), K. Neupane (50), Soniya Shrestha (53), N. Simkhada (57), and A. Dhakal (59).

Images 61–80. 61—Great Eggfly | 62—Danaid Eggfly | 63—Peacock Pansy | 64—Gray Pansy | 65—Yellow Pansy | 66—Chocolate Pansy | 67—Lemon Pansy | 68—Blue Pansy | 69—Banded Treebrown | 70—Common Evening Brown | 71—Dark Evening Brown | 72—White‐line Bushbrown | 73—Dark‐branded Bushbrown | 74—Common Bushbrown | 75—Long‐brand Bushbrown | 76—Plain Sailer | 77—Common Sailer | 78—Jungle Brown | 79—Common Lascar | 80—Glassy Tiger. Photos: P. Chataut (61, 66, 68, 74, and 76), Subarna Shrestha (62, 63, 67, 75, 77, 78, and 80), N. Pradhan (64), K. Thapa (65), M.S. Miya (69 and 72), A. Dhakal (70 and 71), and Soniya Shrestha (73 and 79).

Images 81–100. 81—Tabby | 82—Common Jester | 83—Common Earl | 84—Blue Tiger | 85—Dark Blue Tiger | 86—Vagrant | 87—Indian Red Admiral | 88—Common Five‐ring | 89—Common Four‐ring | 90—Tailed Jay | 91—Common Jay | 92—Common Bluebottle | 93—Common Rose | 94—Common Mime | 95—Lime Swallowtail | 96—Great Mormon | 97—Yellow Helen | 98—Common Mormon | 99—Pioneer | 100—Chocolate Albatross. Photos: A. Neupane (81), M.S. Miya (82, 92, and 100), P. Chataut (83, 89, 90, and 99), K. Thapa (84 and 93), A. Dhakal (85 and 97), K. Neupane (86 and 96), Subarna Shrestha (87, 88, 91, 94, and 95), and Soniya Shrestha (98).

Images 101–115. 101—Common Emigrant | 102—Lemon Emigrant | 103—Mottled Emigrant | 104—Red‐spot Jezebel | 105—Painted Jezebel | 106—Red‐base Jezebel | 107—Three‐spot Grass Yellow | 108—Small Grass Yellow | 109—Common Grass Yellow | 110—Yellow Orange Tip | 111—Psyche | 112—Large Cabbage White | 113—Indian Cabbage White | 114—Bath White | 115—Double‐banded Judy. Photos: A. Dhakal (101), P. Chataut (102, 103, 104, 105, 109, 110, 111, and 114), Subarna Shrestha (106, 107, 113, and 115), K. Thapa (108), and M.S. Miya (112).
